# Management of severe neonatal respiratory distress due to vertical transmission of severe acute respiratory syndrome coronavirus 2: a case report

**DOI:** 10.1186/s13256-022-03364-0

**Published:** 2022-03-28

**Authors:** Anne C. Verheijen, Eva E. R. Janssen, Mayke E. van der Putten, Marieke W. P. van Horck, Gijs T. J. van Well, Inge H. M. Van Loo, Matthias C. Hütten, Karen Van Mechelen

**Affiliations:** 1 Department of Pediatrics, Maastricht University Medical Center, Maastricht, The Netherlands; 2 Division of Neonatology, Department of Pediatrics, Maastricht University Medical Center, P. Debyelaan 25, 6229 HX Maastricht, The Netherlands; 3 Division of Child Pulmonology, Department of Pediatrics, Maastricht University Medical Center, Maastricht, The Netherlands; 4 Zuyderland Medical Center, Heerlen, The Netherlands; 5 Division of Infectiology-Immunology, Department of Pediatrics, Maastricht University Medical Center, Maastricht, The Netherlands; 6 Department of Microbiology, Maastricht University Medical Center, Maastricht, The Netherlands

**Keywords:** Severe acute respiratory syndrome coronavirus 2, Neonate, Respiratory management, Mechanical ventilation, *S. aureus* pneumonia, Case report

## Abstract

**Background:**

Neonates with severe acute respiratory syndrome coronavirus 2 infection are usually asymptomatic or have mild to moderate symptoms. Acute respiratory distress syndrome due to severe acute respiratory syndrome coronavirus 2 with respiratory insufficiency is rare. Therefore, information about the best intensive care strategy for neonates requiring mechanical ventilation is lacking. We report a neonatal case of severe acute respiratory distress syndrome, probably due to vertical transmission of severe acute respiratory syndrome coronavirus 2, complicated by *Staphylococcus aureus* sepsis. We aim to inform pediatric providers on the clinical course and acute management considerations in coronavirus disease-related neonatal acute respiratory distress syndrome.

**Case presentation:**

A late preterm (gestational age 36 0/7 weeks) Caucasian girl was born from a severe acute respiratory syndrome coronavirus 2-positive mother and tested positive for severe acute respiratory syndrome coronavirus 2 at 19 hours after birth. She developed acute respiratory distress syndrome requiring intensive care admission and mechanical ventilation. The clinical course was complicated by *S. aureus* pneumonia and bacteremia. Multimodal management included well-established interventions for respiratory distress syndrome such as surfactant therapy, high-frequency oscillatory ventilation, and inhaled nitric oxide, combined with therapies extrapolated from adult care for severe acute respiratory syndrome coronavirus 2 patients such as dexamethasone, coronavirus disease 2019-specific immunoglobins, and prophylactic low-molecular-weight heparin. The neonate was successfully weaned from the ventilator and improved clinically.

**Conclusion:**

This case shows a rare but serious neonatal severe acute respiratory syndrome coronavirus 2 infection, leading to severe acute respiratory distress syndrome. Because of limited therapy guidelines for neonates, we suggest multimodal management with awareness of the possibility of *S. aureus* coinfection, to treat this age group successful.

## Background

Children with severe acute respiratory syndrome coronavirus 2 (SARS-CoV-2) infection are usually asymptomatic or have mild to moderate signs of infection [1, 2]. Although neonates are more vulnerable to severe infections [1–4], acute respiratory distress syndrome (ARDS) due to SARS-CoV-2 is rare [1, 5, 6]. In a metaanalysis including all cases of neonatal SARS-CoV-2 infections up until 30 August 2020, 52% of neonates presented with respiratory manifestations, but none of them fulfilled the criteria of ARDS [7]. Some papers report a few neonates with ARDS or respiratory insufficiency [8–12]. Nevertheless, detailed neonatal reports are limited, and management considerations are extrapolated from pediatric and adult care. Given that severe neonatal ARDS due to SARS-CoV-2 is rare, we aim to inform pediatric providers on the clinical course and acute management considerations in neonates.

## Case presentation

A 30-year-old pregnant Caucasian woman without significant past medical history tested positive for SARS-CoV-2 by polymerase chain reaction (PCR) in the nasopharyngeal swab. Four days later, she developed malaise, fever, and rhinorrhea without need for respiratory support or any other therapeutic intervention. At that time, vaccination was not yet possible in the Netherlands. Seven days after the positive test, at a gestational age of 36 0/7 weeks, she gave birth by emergency cesarean section because of fetal distress. Both parents wore surgical masks, but there was a short moment of unprotected mother–child contact directly after birth. Afterward, the parents and patient were strictly separated. Parents were allowed to visit wearing FFP-2 masks if they had no clinical signs of active SARS-CoV-2 infection. A female neonate, with birth weight of 2660 g (10th–50th percentile), was born. The Apgar score was 4 and 7, at respectively 1 and 5 minutes. Inflation breaths and continuous positive airway pressure (CPAP) were given. The girl was cared for in isolation according to the local protocol. None of the staff caring for the patient tested positive for SARS-CoV-2 during this period. Because of progressive respiratory insufficiency with fraction of inspired oxygen (FiO_2_) of 1.0, the neonate was intubated and endotracheal surfactant (twice 100 mg/kg) was administered. Thereafter, the girl was transferred to a tertiary Neonatal Intensive Care Unit. The nasopharyngeal aspirate, obtained 19 hours after birth, and the endotracheal aspirate tested positive for SARS-CoV-2 by PCR. Viral genome sequencing demonstrated a strain that was common in the Netherlands (20E (EU1) clade (Pangolin lineage B.1.177)), and strains from mother and child were identical. Cycle threshold values decreased minimally over the first 5 days, reflecting an increasing viral load (Table 1) [13]. Furthermore, there was lymphopenia and slightly elevated C-reactive protein. SARS-CoV-2 total immunoglobulins (Ig) were negative on day of life (DOL) 1 (both Ig and IgM) and became positive on DOL 9. Laboratory evaluations are presented in Table 1. No amniotic fluid, cord blood, or placenta was available for diagnostic testing as they were not stored in the peripheral hospital where the girl was born.

**Table 1:** Laboratory results during hospitalization

Laboratory test	Reference range	DOL 1	DOL 2	DOL 5	DOL 8	DOL 9	DOL 10	DOL 11	DOL 12
Hemoglobin (mmol/L)	9.5–13	10.6	12.1	9.5	11.0	9.7	–	8.7	–
Total leukocyte count (× 10^9^/L)	10–25	20.4	9.7	4.5	7.8	8.2	–	7.8	–
Total lymphocyte count (× 10^9^/L)	2–12.6	7.7	–	1.6	–	4.3	–	–	–
Platelet count (× 10^9^/L)	150–350	> 244	176	89	64	110	–	164	–
C-reactive protein (mg/L)	< 10	< 4	2	22	1	1	2	51	27
Ferritin (µg/L)	200–600	–	–	–	–	–	2379	1764	1057
Aspartate aminotransferase (U/L)	< 31	–	–	–	–	–	66	–	–
Lactate dehydrogenase (U/L)	150–380	–	–	–	–	–	1364	838	–
d-dimer (µg/L)	< 500	–	–	–	–	–	5147	1286	–
Fibrinogen (g/L)	1.25–3.0	–	–	–	–	–	2.2	3.7	–
Prothrombin time (s)	11–14.5	–	–	–	–	–	11.2	–	–
SARS-CoV-2 total Ig	Neg–Pos	Neg	–	–	–	Pos	–	–	–
SARS-CoV-2 IgM	Neg–Pos	Neg	–	–	–	Pos	–	–	–
Cycle threshold (upper respiratory tract)		26	–	–	25	–	–	–	–
Cycle threshold (sputum)		–	–	13	–	–	23	–	–

–, not available; Ig, immunoglobulin

*DOL*: Day of life; *SARS-CoV-2*: Severe acute respiratory distress syndrome coronavirus 2

The neonate required mechanical ventilation with maximum positive inspiratory pressure (PIP) of 28 cmH_2_O and fraction of inspired oxygen (FiO_2_) of 0.33 for 5 days. On DOL 5, she was extubated and received CPAP with FiO_2_ of 0.3–0.4. During the first week of life, she had intermittent fever and received broad-spectrum antibiotics for 5 days to treat suspected early-onset sepsis. Blood culture remained negative. Furthermore, she received a combination of parenteral nutrition and formula feeding through a nasogastric tube. Breast milk was not possible during the first 5 days of life because of maternal use of opioids.

On DOL 9, the neonate was reintubated because of oxygenation failure (CPAP with PEEP of 8 cmH_2_O and FiO_2_ of 0.9). Computed tomography (CT) of the chest showed bilateral areas of consolidation with coexisting patchy ground-glass opacities. There were no signs of congenital interstitial lung diseases or central pulmonary embolisms. The CT was rated as CO-RADS 5 (COVID-19 Reporting and Data System) with a CT severity scale of 18. This implies a very high level of suspicion for pulmonary involvement by COVID-19 [14]. Since there was an oxygenation index between 14–22, the criteria for neonatal ARDS were met [15]. Because secondary surfactant deficiency is associated with ARDS, endotracheal surfactant was administrated twice more (100 mg/kg) with short-time effects. To overcome ventilation–perfusion mismatch, inhaled nitric oxide (iNO) at 20 parts per million was given (DOL 9–18). Following established adult SARS-CoV-2 intensive care unit protocols, prone positioning was performed, dexamethasone intravenously (IV) (DOL 9–26) (starting with 0.150 mg/kg [16] and gradually decreased), flucloxacillin IV empirically, and prophylactic subcutaneous low-molecular-weight heparin (DOL 10–32) were started (325 IE/day). After consulting a pediatric immunologist and experts of the Dutch national blood transfusion laboratory (Sanquin), coronavirus disease (COVID-19)-specific IgG were administrated IV on DOL 11 because of the negative IgG levels on DOL 1 and the critical condition of the neonate. No antiviral drugs or inotropes were given.

On DOL 11, there was further clinical deterioration, with high oxygen requirement on conventional ventilation, so high-frequency oscillatory ventilation (HFOV) was started with good effect (maximum mean airway pressure of 18 cmH_2_O, amplitude 37%). The oxygenation index varied between 12 and 22. X-ray of the thorax showed slightly progressive infiltrative changes, now more appearing as consolidations. Blood culture was positive for *Staphylococcus aureus* (*S. aureus*). The diagnosis of *S. aureus* pneumonia with bacteremia was established. High-dose flucloxacillin IV was given for 20 days. On DOL 12, the girl developed a pneumothorax, requiring a chest tube. Thereafter, there was a gradual improvement of the respiratory function. On DOL 18, HFOV was ceased and conventional mechanical ventilation was restarted. On DOL 22, she was extubated and respiratory support was continued with heated humidified high-flow nasal cannula with flow of 8 L/min and FiO_2_ of 0.6. A short course of prednisolone was given (0.53 mg/kg during 7 days). Repeated SARS-CoV-2 PCR testing remained positive in the nasopharyngeal aspirate up to DOL 31. Afterward, the tests were negative. In the following weeks, respiratory support and supplemental oxygen could be reduced. She is now 11 months old and does not have major respiratory complaints. She uses an inhalation corticosteroid and salbutamol because of bronchus obstruction during periods of respiratory infections. She receives tube feeding because of feeding difficulties. Her motoric and neurological development is within normal ranges.

**Fig. 1:**
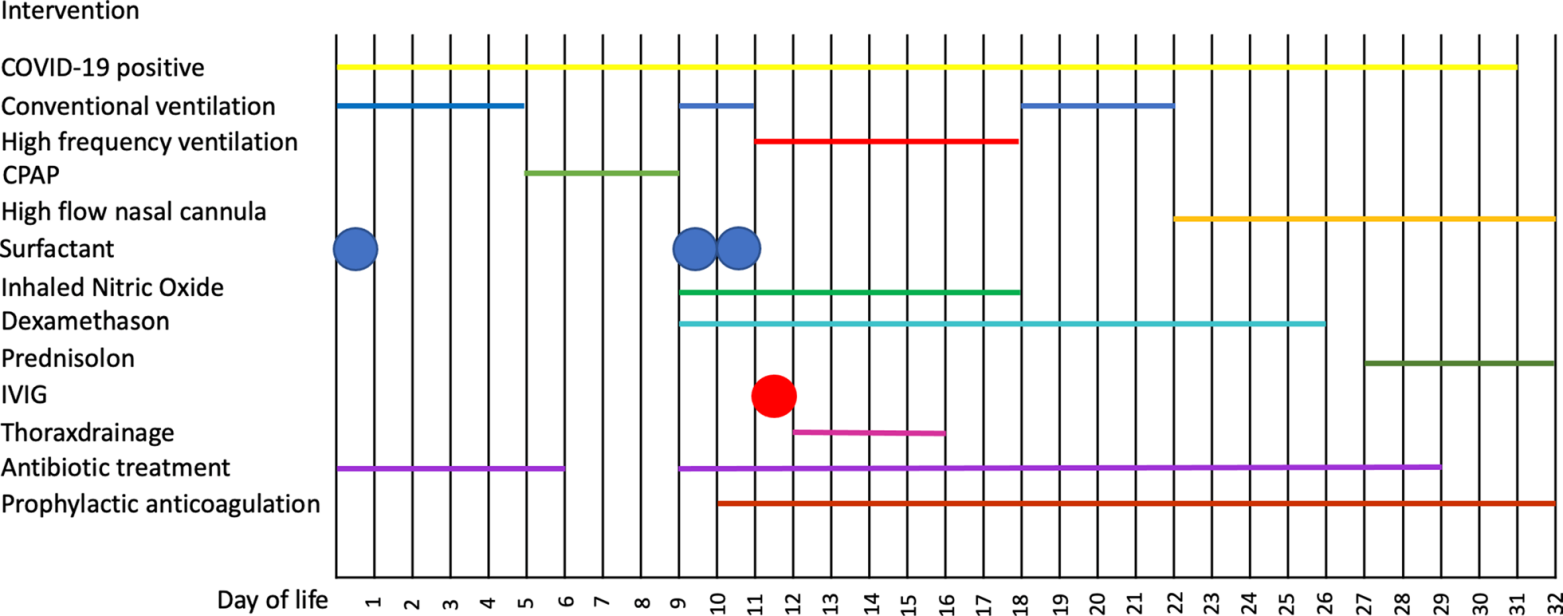
Overview of clinical course and therapeutic interventions

An overview of the clinical course and therapeutic interventions is presented in Fig. 1.

## Discussion

Neonatal ARDS due to SARS-CoV-2 infection is rare. In the Netherlands, 5105 neonates were born to women infected with SARS-CoV-2 between March 2020 and 7 December 2021. Of them, two patients tested positive for SARS-CoV-2 directly postpartum, but our patient is the only one to have developed respiratory insufficiency [17]. In the German COVID-19-Related Obstetric and Neonatal Outcome Study (CRONOS) registry, nine neonates from mothers who tested positive for SARS-CoV-2 between April 2020 and 27 November 2020 also tested positive. Seven of them tested positive within 48 hours but were asymptomatic. Two infants tested positive after 48 hours, with one of them requiring mechanical ventilation [18]. In a metaanalysis by Rashetti *et al*., including all cases of neonatal SARS-CoV-2 infections up until 30 August 2020, none of the 176 infected neonates had ARDS [7]. However, some articles report neonates with ARDS due to SARS-CoV-2 [8–12]. Cohort studies in the UK [10] and Europe [11, 12] reported that respectively 3 of 66 and 4 of 35 SARS-CoV-2-positive neonates required mechanical ventilation. Of these seven neonates, two were born premature and two had underlying cardiac anomalies [10, 11].

When comparing literature on the clinical course and management of pediatric and adult SARS-CoV-2 infection with our case, there are many similarities but also differences. First, lymphopenia, as in our case, is rare in children and neonates, but is observed in adults with more severe disease [19]. The clinical deterioration on DOL 9 [20] is in concordance with adult literature, as are the chest CT findings [4]. According to the Montreux definition, neonatal ARDS is respiratory distress with acute onset from a known or suspected clinical insult and with diffuse, bilateral, and irregular opacities, infiltrates, or complete opacification of the lungs, which cannot be fully explained otherwise [15]. An oxygenation index above 16 is categorized as severe ARDS [15]. Our patient fulfilled the criteria of the Montreux definition for neonatal severe ARDS. Nevertheless, it is difficult to differentiate whether the patchy opacity appearance on the CT scan in our case was secondary to the *S. aureus* pneumonia/bacteremia, the “cytokine storm” phase of SARS-CoV-2 infection, atelectasis, or a combination of factors. It is suggested that the start of ARDS was caused by SARS-CoV-2 infection, with a further deterioration on DOL 11 due to *S. aureus* coinfection. *S. aureus* is a known pathogen to cause coinfection in SARS-CoV-2 [21].

There is almost no information on neonatal COVID-19 therapies. According to a systematic review by Raba *et al*., half of the children younger than 1 year received medications: 48% were given interferon, 36% antibiotics, 8% steroids, and 7% immunoglobulins [4]. In our patient, endotracheal surfactant was administrated with short-time positive effects, which were also reported by Correia *et al*. [8] and Trieu *et al*. [9]. Surfactant administration is common practice for preterm neonates or neonates with secondary surfactant deficiency due to meconium aspiration syndrome or inflammatory disorders. Secondary surfactant deficiency in these disorders and in ARDS is probably due to secretory phospholipase A2, which triggers the inflammatory cascade and hydrolyses surfactant phospholipids [22]. Furthermore, like other respiratory viruses, SARS-CoV-2 damages type II pneumocytes, which secrete pulmonary surfactants [23]. When considering this pathophysiology, surfactant administration potentially contributes positively in treating adults with ARDS also [23–25]. Furthermore, dexamethasone IV was given because of severe ARDS. The World Health Organization (WHO) strongly recommends corticosteroids for the treatment of adults with severe COVID-19, but the applicability in children and neonates is uncertain as they were underrepresented in clinical trials [26]. Nevertheless, dexamethasone treatment is frequently used in preterm neonates to facilitate extubation and prevent bronchopulmonary dysplasia [27]. Our patient received empirical antibiotics directed to respiratory pathogens, such as *S. aureus*, which is supported by literature [28, 29]. It is probable that the deterioration on DOL 9 in our patient was due to COVID-19 and was complicated by the *S. aureus* pneumonia [28].

Prophylactic low-molecular-weight heparin was started empirically, according to adult and pediatric guidelines [29]. Furthermore, HFOV and iNO were used as rescue therapies with good effects. These effects were also observed by Correia *et al*. and Trieu *et al*. in neonates with severe ARDS due to SARS-CoV-2 [8, 9]. In adults, HFOV does not reduce mortality [30], but no studies in COVID-19 patients have been done. Some reviews show promising results of iNO in adults with COVID-19, but its safety and effectiveness need further evaluation [31, 32]. Finally, antivirals, immunomodulators, and other adjunctive therapies are not recommended by the WHO as studies show no clear benefit [29]. Nevertheless, immunomodulating therapy could be beneficial for children with severe COVID-19 [33]. Some cases describe possible positive effects of plasma administration [9, 34] and immunoglobulins in neonates [35, 36], as in our case. At the moment, no randomized controlled trials in children are available.

Besides the differences in the clinical picture and management, the mode of transmission of SARS-CoV-2 also differs between neonates and other patients. Neonates can be infected by vertical transmission, either congenitally or intrapartum, or by horizontal transmission postnatally [1, 7, 37]. Earlier systematic reviews and metaanalyses suggest that vertical transmission is unlikely [38–40]. However, more recent case series report possible vertical transmission [8, 35, 41–45]. Based on the classification published by Shah *et al*. [37], we classify our patient as having a confirmed neonatal infection acquired intrapartum, although congenital infection cannot be excluded. Probably, our patient is a vertically transmitted case because the endotracheal aspirate was already positive for SARS-CoV-2 within 19 hours. The short moment of unprotected mother–child contact cannot explain this positivity so fast after birth, as the incubation period of SARS-CoV-2 is 2–14 days [46].

Finally, although long-term sequelae of SARS-CoV-2 infection in neonates are not described, follow-up for the known consequences in adults is necessary.

## Conclusion

Neonatal SARS-CoV-2 infection with severe ARDS is rare. Nevertheless, clinicians need to include SARS-CoV-2 in the differential diagnosis of neonatal ARDS. Because of the limited therapy guidelines for neonates, we suggest a multimodal management including surfactant, high-frequency oscillation ventilation, inhaled nitric oxide, dexamethasone, and prophylactic low-molecular-weight heparin. Awareness of *S. aureus* coinfection, also in this patient group, is important for successful treatment.

## Data Availability

All data generated or analyzed during this study are included in this article. Further inquiries can be directed to the corresponding author.

## Abbreviations

ARDS: Acute respiratory distress syndrome
COVID-19: Coronavirus disease 2019
CPAP: Continuous positive airway pressure
CRONOS: COVID-19-Related Obstetric and Neonatal Outcome Study
CT: Computed tomography
DOL: Day of life
FiO_2_: Fraction of inspired oxygen
HFOV: High-frequency oscillatory ventilation
Ig: Immunoglobulin
iNO: Inhaled nitric oxide
IV: Intravenously
PIP: Positive inspiratory pressure
PCR: Polymerase chain reaction
*S.*: *Staphylococcus*
SARS-CoV-2: Severe acute respiratory distress syndrome coronavirus 2
WHO: World Health Organization
